# Improvement of the thermoelectric properties of a MoO_3_ monolayer through oxygen vacancies

**DOI:** 10.3762/bjnano.10.199

**Published:** 2019-10-25

**Authors:** Wenwen Zheng, Wei Cao, Ziyu Wang, Huixiong Deng, Jing Shi, Rui Xiong

**Affiliations:** 1 Hubei Key Laboratory of Optical Information and Pattern Recognition, School of Mathematics and Physics, Wuhan Institute of Technology, Wuhan 430205, China; 2 Key Laboratory of Artificial Micro- and Nano-Structures of Ministry of Education, School of Physics and Technology, Wuhan University, Wuhan 430072, China; 3 The Institute of Technological Sciences, Wuhan University, Wuhan 430072, China; 4 State Key Laboratory of Superlattices and Microstructures, Institute of Semiconductors, Chinese Academy of Sciences, P. O. Box 912, Beijing 100083, China

**Keywords:** Boltzmann transport theory, first-principles calculations, molybdenum trioxides, MoO_3_ monolayer, oxygen vacancies, thermoelectric properties

## Abstract

We have investigated the thermoelectric properties of a pristine MoO_3_ monolayer and its defective structures with different oxygen vacancies using first-principles methods combined with Boltzmann transport theory. Our results show that the thermoelectric properties of the MoO_3_ monolayer exhibit an evident anisotropic behavior which is caused by the similar anisotropy of the electrical and thermal conductivity. The thermoelectric materials figure of merit (*ZT*) value along the *x*- and the *y*-axis is 0.72 and 0.08 at 300 K, respectively. Moreover, the creation of oxygen vacancies leads to a sharp peak near the Fermi level in the density of states. This proves to be an effective way to enhance the *ZT* values of the MoO_3_ monolayer. The increased *ZT* values can reach 0.84 (*x*-axis) and 0.12 (*y*-axis) at 300 K.

## Introduction

Thermoelectric materials that can directly convert temperature gradients to voltage gradients and vice versa provide a valid strategy to mitigate the global energy crisis. Owing to the unique ability of utilizing waste heat without generating any greenhouse gas, thermoelectric technology has attracted increasing attention [1]. Nevertheless, the application of thermoelectric materials is limited by the low energy conversion efficiency. The performance of thermoelectric materials is usually measured by a figure of merit (*ZT*) defined as *ZT* = *S*^2^σ*T*/κ, where *S*, σ, *T* and κ represent the Seebeck coefficient, electrical conductivity, temperature and thermal conductivity, respectively [2–3]. In the past decade, great efforts have been made to boost the capabilities of thermoelectric materials. Unfortunately, the application of conventional thermoelectric materials is still limited by inefficiency and problems with high-cost, stability and toxicity.

As promising candidates to address these severe challenges, transition metal oxides (TMOs) provide a vast variety of low-cost and environmentally friendly materials. From insulating to semiconducting and conducting, TMOs exhibit wide-ranging electrical and magnetic characteristics that depend on their geometric structure, doping concentration and stoichiometry ratio [4]. TMOs have been used in many fields such as Li-ion batteries, electrochemical capacitors and fuel cells [5]. Meanwhile, the thermoelectric application of TMO-based materials has been explored and their poor efficiency is still the major difficulty [6].

Among the TMOs, layered molybdenum trioxide (MoO_3_) has attracted attention as a potential electrode material in electrochemical products [7] and Li-ion batteries [8–11]. Like most TMOs, bulk MoO_3_ has a wide band gap (about 3.0 eV) and low electrical conductivity, which seems inappropriate for thermoelectric devices. However, the electrical properties (including band gap and conductivity) of MoO_3_ are strongly dependent on the concentration of O vacancy concentrations. Hydrogen-ion intercalation [12] and solar-light irradiation [13] can turn MoO_3_ into MoO_3−_*_x_* and hence increase the electrical conductivity. Understanding the effect of O vacancies in MoO_3_ is very beneficial for its thermoelectric applications. Moreover, low-dimensional materials show a better thermoelectric performance than bulk materials [14]. Few-layer MoO_3_ nanosheets have already been experimentally synthesized by exfoliation similar to graphene [12–13,15–16]. Theoretical research has proved that few-layer MoO_3_ possesses a markedly high carrier mobility above 3000 cm^2^·V^−1^·s^−1^ [17]. Therefore, it is of profound significance to explore the thermoelectric properties of MoO_3_ monolayers and discuss the effect of O vacancies on it.

## Computational methods

In this work, we evaluate the thermoelectric properties of a MoO_3_ monolayer by Boltzmann transport theory and first-principles calculations. The calculations of the electrical properties of the MoO_3_ monolayer are performed using density functional theory (DFT) as implemented in the Vienna ab initio simulation package (VASP) code [18–19]. We utilize the generalized gradient approximation (GGA) of the Perdew–Burke–Ernzerhof (PBE) [20] pseudopotentials without spin–orbit interaction. Since the PBE functional fails to capture the electrical and optical properties of bulk MoO_3_ by a large margin (band gap of less than half of the experimental value), we also employ the Heyd–Scuseria–Ernzerhof (HSE06) hybrid functional [21–22] to obtain a more accurate band structure. The vacuum distance is set to 15 Å to avoid interactions between the MoO_3_ monolayer and its periodic images. A plane-wave basis set with a cutoff of 520 eV is chosen, and the *k*-mesh is tested to be 10 × 10 × 1 for the purpose of convergence.

Based on the electronic structure, the electrical transport properties can be obtained using the Boltzmann transport theory and the constant scattering time approximation as implemented in the BoltzTraP code [23]. To get reliable electrical transport coefficients, a denser *k*-point mesh of 40 × 40 × 1 is used to obtain converged results. Based on the framework of Boltzmann transport theory, the electrical conductivity, σ, and the Seebeck coefficient, *S*, can be expressed as:

[1] $$\sigma =e^{2}\int \text{d}\varepsilon \left(-\frac{\partial f_{0}}{\partial \varepsilon }\right)\;\Sigma \left(\varepsilon \right),$$

[2] $$S=\frac{ek_{\text{B}}}{\sigma }\int \text{d}\varepsilon \left(-\frac{\partial f_{0}}{\partial \varepsilon }\right)\;\Sigma \left(\varepsilon \right)\frac{\varepsilon -u}{k_{\text{B}}T},$$

[3]
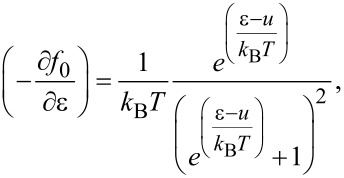


where the *u* is the chemical potential (corresponding to the carrier concentration), *k*_B_ is the Boltzmann constant, *e* is the electron charge and *T* is the absolute temperature. Σ(ε) is the so-called transport distribution function [24]:

[4]
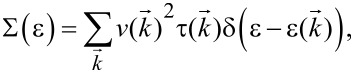


where 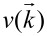 is the group velocity of the carriers and 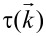 is the relaxation time. The thermal conductivity κ*_e_* is obtained by the Wiedemann–Franz law: κ*_e_* = *L*σ*T*, where *L* is the Lorenz number. For the calculation of the relaxation time τ, we apply the deformation potential (DP) theory [25] where τ is estimated by τ = μm*/*e*. The carrier mobility μ_2D_ in 2D materials is given by


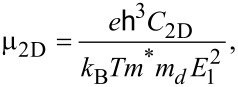


Where m* is the effective mass and *m_d_* is the density of states (DOS) mass determined by


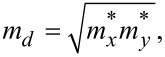


*E*_1_ is the DP constant and *C*_2D_ is the elastic modulus. All parameters corresponding to the carrier mobility and the effective mass are taken from [17] and summarized in Table 1.

**Table 1:** The effective mass (m*), carrier mobility (μ) and relaxation time (τ) along the *x*- and the *y*-directions of the MoO_3_ monolayer at 300 K.

carriers	m_x_*/*m*_0_	m_y_*/*m*_0_	µ*_x_* (cm^2^·V^−1^·s^−1^)	µ*_y_* (cm^2^·V^−1^·s^−1^)	τ*_x_* (ps)	τ*_y_* (ps)

PBE						
electron	0.873	0.594	1608.81	37.52	0.786	0.012
hole	2.064	0.974	800.57	25.56	0.925	0.013
HSE06						
electron	1.056	0.588	793.59	33.60	0.469	0.011
hole	1.669	0.910	396.35	19.99	0.370	0.010

As for phononic transport properties, we calculate the thermal conductivity of the lattice using the Boltzmann transport theory as implemented in the Quantum ESPRESSO (QE) package [26–27] and the ShengBTE code [28]. The pseudopotential files are obtained from the standard solid-state pseudopotentials library [29] and the kinetic energy cutoff is set to 80 Ry. Density functional perturbation theory (DFPT) is adopted from the QE package for the calculation of second-order (harmonic) interatomic force constants (IFCs), and third-order (anharmonic) IFCs are obtained from the ShengBTE code [28] with the maximum atomic interaction distance of the sixth neighbor in the 4 × 4 × 1 supercell. Here, a dense phonon *q*-grid of 52 × 52 × 1 is used to compute the thermal conductivity of the lattice of MoO_3_, and the scale parameter for smearing is set to 0.1.

## Results and Discussion

**Figure 1:**
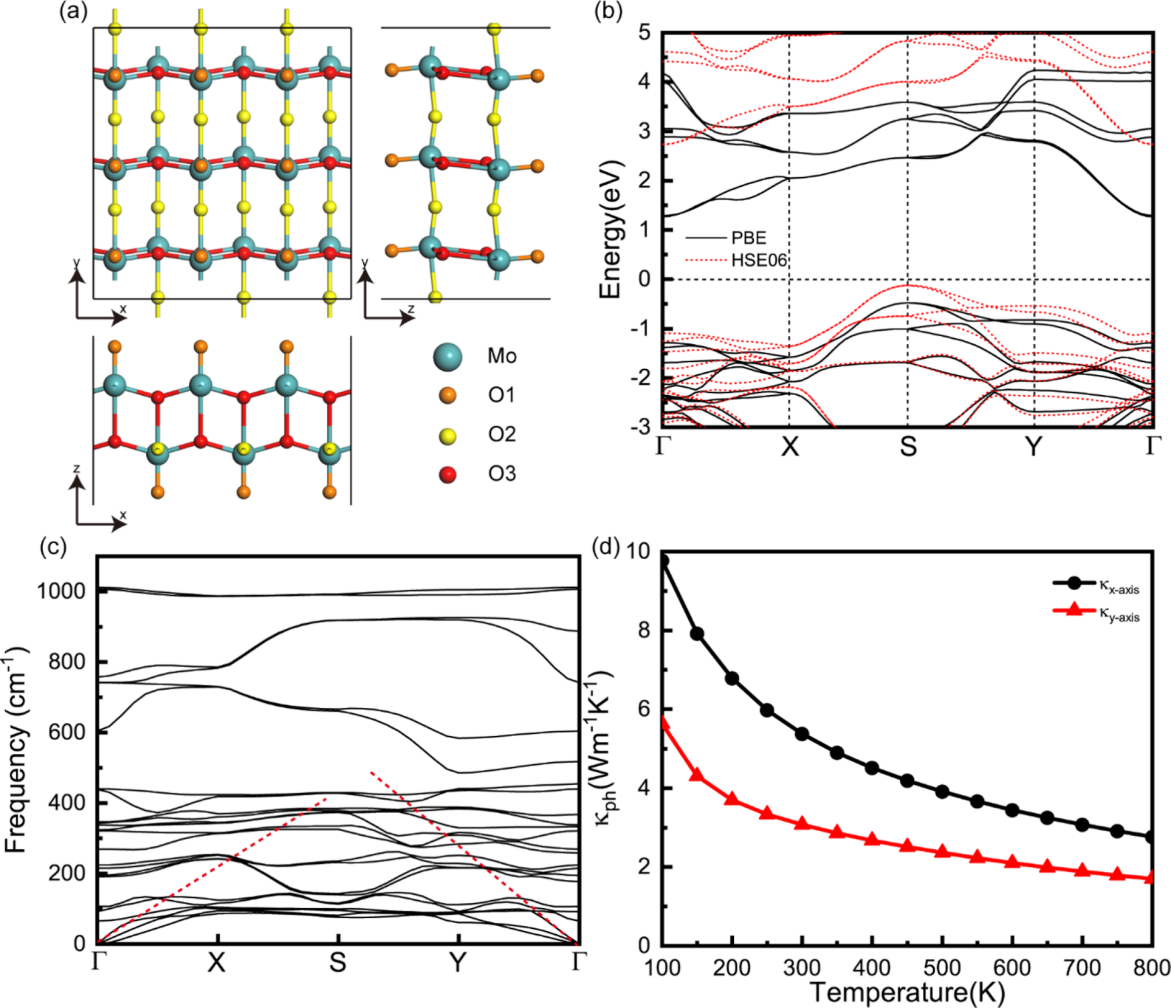
(a) Crystal structures of the MoO_3_ monolayer (3 × 3 × 1 supercell): top and side views. (b) The electronic band structure and (c) the phonon dispersion of the MoO_3_ monolayer along the high-symmetry path. (d) The lattice thermal conductivity, κ_ph_, of the MoO_3_ monolayer along different directions as a function of temperature.

As shown in Figure 1a, the MoO_3_ monolayer is cut from an orthorhombic α-MoO_3_ bulk structure (space group *Pbnm*), and the primitive cell contains eight atoms. Apparently, there are three distinguishable O atoms, that are connected to different molybdenum atoms, as indicated by different colors in Figure 1a. The relaxed lattice constants of the MoO_3_ monolayer are *a* = 3.68 Å and *b* = 3.93 Å, which are similar to the bulk experimental data of 3.70 and 3.96 Å [30]. In Figure 1b, we give the electronic band structure of the MoO_3_ monolayer as calculated using the PBE potential and the HSE06 hybrid functional potential. One can see clearly that both of the two different potentials yield similar electronic band structures for the larger energy gaps. The minimum point of the first conduction band (CBM) appears at the Γ point, while the maximum of the first valence band (VBM) appears at the S point. The indirect bandgap of the MoO_3_ monolayer is computed as 1.79 eV for PBE and 2.85 eV for HSE06, which is consistent with previous studies [17,31]. Since the MoO_3_ monolayer is a wide-gap semiconductor, it is likely to exhibit a giant Seebeck coefficient and low electrical conductivity. The discrepancies between the results obtained using the PBE potential and the HSE06 hybrid functional are inconspicuous concerning the Seebeck coefficient and the electrical conductivity. Considering the extremely slow speed of the HSE06 hybrid functional potential, we use the PBE potential to get the electrical transport properties of the MoO_3_ monolayer in the following work.

The phonon dispersion in the high-symmetry directions of the first Brillouin zone of the MoO_3_ monolayer is plotted in Figure 1c. There is no imaginary frequency, ensuring the stability of the MoO_3_ monolayer. It is well known that the acoustical phonon branches possess large group velocities and mainly determine the thermal conductivity of the lattice [32]. As the dashed lines emphasize in Figure 1c, the acoustic phonon group velocity along the Γ–Y direction is larger than that along the Γ–X direction, which is an indication of anisotropic phonon transport. The thermal conductivity of the lattice, κ_ph_, along different directions as a function of temperature is depicted in Figure 1d. It is obvious that the thermal transport properties of the MoO_3_ monolayer show an anisotropic behavior, where the thermal conductivity of the lattice along the *x*-axis is higher than that along the *y*-axis. At room temperature, κ_ph_ is 5.38 and 3.07 W·mK^−1^ along the *x*- and *y*-direction, respectively. As a result of the intrinsic enhancement of the phonon–phonon scattering with temperature, the thermal conductivity of the lattice of the MoO_3_ monolayer decreases gradually with increasing temperature following a 1/*T* dependence like most crystalline materials.

**Figure 2:**
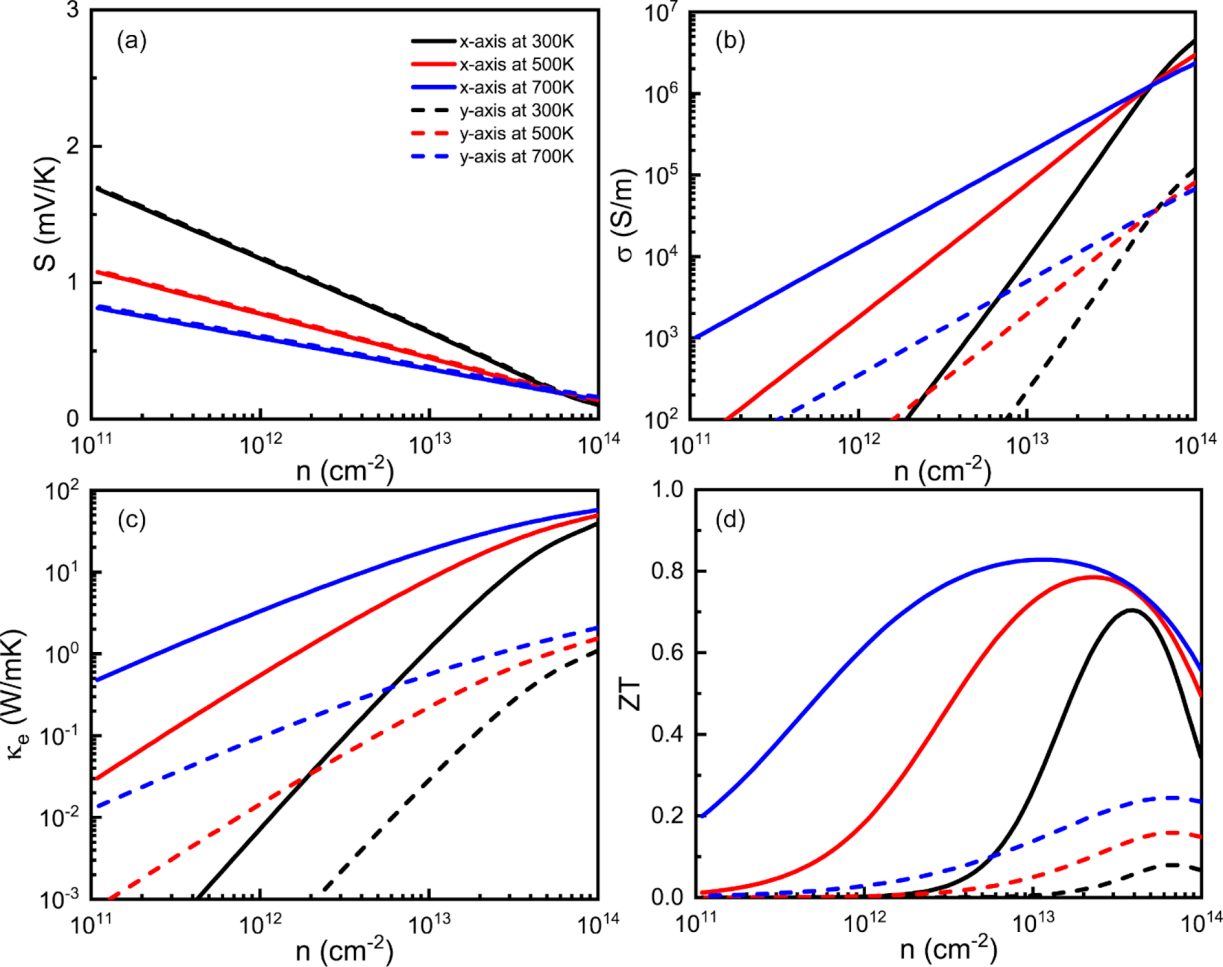
Transport and thermoelectric properties of the MoO_3_ monolayer. (a) Seebeck coefficient, *S*, (b) electrical conductivity, σ, (c) electrical conductivity, κ*_e_*, and (d) *ZT* values as a function of the carrier concentration.

In Figure 2a–c, we demonstrate the electrical transport properties of the MoO_3_ monolayer as a function of the carrier concentration at three typical temperatures (*T* = 300, 500 and 700 K). Note that the *n*-type and *p*-type transport properties of MoO_3_ monolayer are uniform. Therefore, only *p*-type cases are shown here. As shown in Figure 2a, the Seebeck coefficient *S* of the MoO_3_ monolayer has the same value along the *x*- and *y*-directions. While the temperature increases from 300 to 700 K, the Seebeck coefficient of the MoO_3_ monolayer decreases gradually according to the relation: *S* ~ (1/*n*)^2/3^ [3]. The maximum value of *S* calculated for the MoO_3_ monolayer is 1.69 mV·K^−1^ at room temperature. Such a large *S* value can be explained by the proportionality of the Seebeck coefficient to the bandgap [33]. Unlike the Seebeck coefficient, the electrical and thermal conductivities exhibit a clear anisotropic behavior which is attributed to the anisotropic relaxation time [17]. Figure 2b reveals that the electrical conductivity along the *x*-axis is obviously much higher than along the *y*-axis. For example, we compute σ = 8268 S/m along the *x*-axis, while only σ = 338 S/m along the *y*-axis at room temperature and *n* = 10^13^ cm^−2^. The thermal conductivity of the MoO_3_ monolayer also exhibits an evident anisotropic behavior and increases gradually with temperature, as shown in Figure 2c. Particularly, κ*_e_* = 1.29 and 0.032 W·mK^−1^ along the *x*- and *y*-axis at room temperature and *n* = 10^13^ cm^−2^. By combining the phononic and electrical transport properties presented above, we can obtain the *ZT* value of the MoO_3_ monolayer as a function of the carrier concentration, as presented in Figure 2d. It is found that the *ZT* value is much higher along the *x*-axis than along the *y*-axis. The maximum *ZT* value along the *x*-axis reaches 0.84 at 700 K which is nearly four times larger than the corresponding value along the *y*-axis. The carrier concentration needed to attain the maximum *ZT* value in the *n*-type MoO_3_ monolayer is about 1.16 × 10^13^ cm^−2^ (*x*-axis) and 6.67 × 10^13^ cm^−2^ (*y*-axis).

In order to improve the thermoelectric performance of the MoO_3_ monolayer, we decided to introduce an O vacancy. Therefore, we remove one O atom from the 3 × 3 × 1 supercell (72 atoms) to construct a neutral defect, such that the stoichiometric proportion is MoO_2.94_. Since there are three types of O atoms in the primitive cell, we named the three defective structures V_O1_, V_O2_ and V_O3_. Further details of the defect structures are presented in Supporting Information File 1. The formation energy, *E*_f_, of a neutral defect is defined as: *E*_f_ = *E*_tot_(defect) − *E*_tot_(supercell) + ½ *E*_tot_(O_2_), where *E*_tot_(defect) is the total energy of the supercell containing the defect, *E*_tot_(supercell) is the total energy of the perfect supercell and *E*_tot_(O_2_) is the total energy of the oxygen molecules. Then we calculate the formation energies and band gaps of these defect structures. The absolute *E*_f_ values are 2.074 eV (V_O1_), 2.076 eV (V_O2_) and 4.108 eV (V_O3_) while the band gaps are 0.837 eV (V_O1_), 0.797 eV (V_O2_) and 0.831 eV (V_O3_). In contrast to the bulk MoO_3_, the three defective structures finally lead to only two stable structures after relaxation which is attributed to the absence of interlayer van der Waals forces.

**Figure 3:**
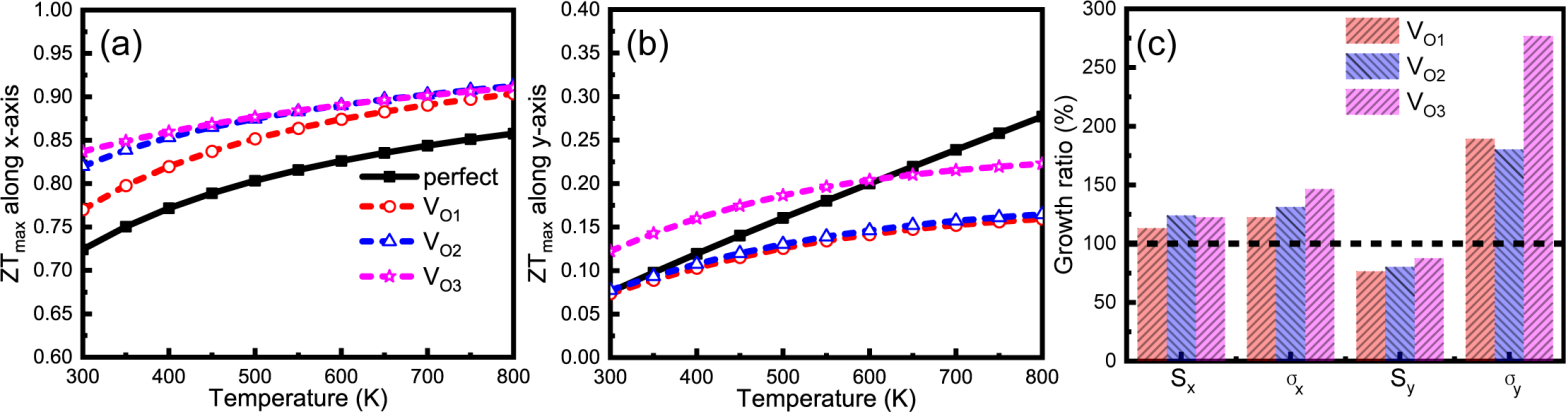
The maximum *ZT* values as a function of temperature along (a) the *x*-axis and (b) the *y*-axis. (c) The growth ratio of the Seebeck coefficient and the electrical conductivity along the *x*-axis and the *y*-axis at room temperature.

It is difficult to calculate the thermal conductivity of the lattice and electrical relaxation time of a 71 atom primitive cell employing Boltzmann transport theory. Therefore, we assume that the thermal conductivity of the lattice and electrical relaxation time remain unchanged. In fact, O vacancies can result in the reduction of the thermal conductivity of the lattice due to anisotropic phonon scattering at the vacancies and the final *ZT* values will be higher. The structures exhibit strong Mo–O3–Mo chains along the *x*-axis and Mo–O2–Mo chains along the *y*-axis. The anisotropic thermal conductivity of the vacancy-induced MoO_3_ monolayer can be modified by controlling the O vacancy [34]. For instance, the O3 vacancy mainly disrupts the Mo–O3–Mo chain and reduces the phonon transport along the *x*-axis, which will result in a lower thermal conductivity of the lattice along the *x*-axis than along the *y*-axis. For briefness and clarity, we display the maximum *ZT* values in Figure 3a and Figure 3b. Note that when vacancies are induced, the *ZT* values along the *x*-axis increase immediately, especially in the low-temperature region. However, the behavior in the *y*-direction is considerably different. In Figure 3b, the *ZT* values along the *y*-axis are obviously higher for V_O3_ than in the other two cases at usual working temperatures of 300–600 K. The conclusion is that inducing the O vacancy at the O3 position (V_O3_) yields the largest enhancement of the thermoelectric properties of the MoO_3_ monolayer. The maximum *ZT* values along the *x*- and the *y*-axis are 0.83 and 0.12, respectively, at room temperature. This indicates that the defective MoO_3_ monolayer is indeed a promising candidate for good thermoelectric materials. We take the results at room temperature as an example and calculate the growth ratio of the Seebeck coefficient and the electrical conductivity along the *x*- and the *y*-axis in Figure 3c. It is found that the Seebeck coefficient and the electrical conductivity along the *x*-axis increase simultaneously, which results in higher *ZT* values, as shown in Figure 3a. However, the results are very different concerning the *y*-direction. The electrical conductivity along the *y*-axis increases faster than that along the *x*-axis. This leads also to a much lower Seebeck coefficient. As a consequence, the *ZT* values along the *y*-axis are not satisfactory compared with the corresponding values along the *x*-axis.

**Figure 4:**
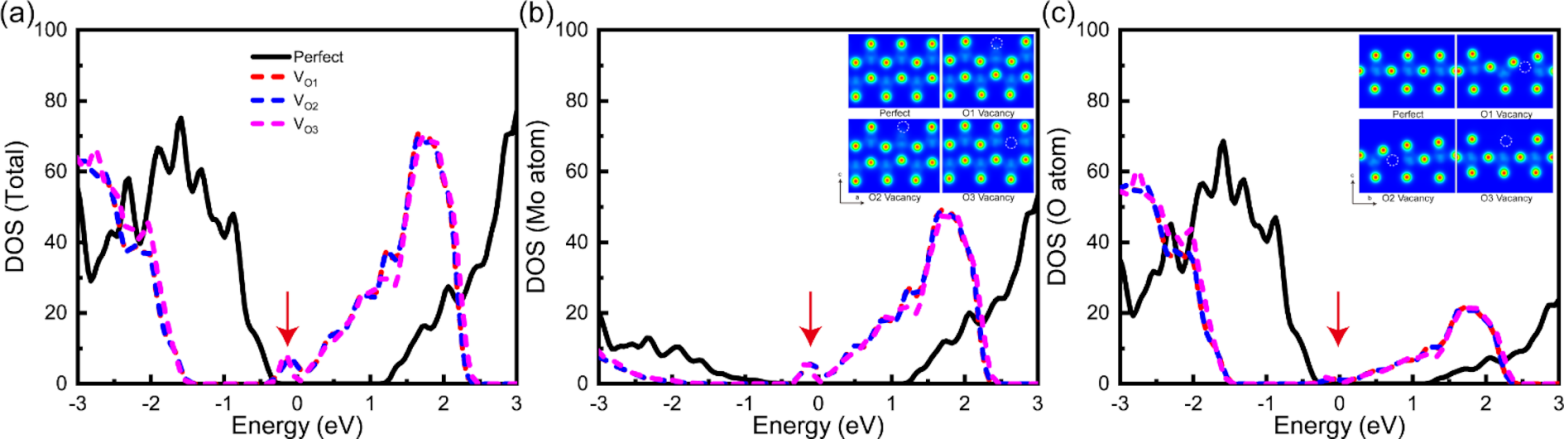
Calculated (a) total electronic density of states (DOS) and projected DOS for (b) Mo and (c) O atoms of the MoO_3_ monolayer. The insets of (b) and (c) show the total charge density from different views.

To give a clear reason for the strengthening of the thermoelectric properties, we show the electronic DOS of these structures in Figure 4. Upon the introduction of the O vacancy, the Fermi level moves closer to the conduction band and an asymmetric sharp peak occurs around the bottom of the conduction band, as highlighted in Figure 4a. The sharp peak in the DOS mainly comes from the Mo atom indicated by the projected DOS of Mo and O atoms presented in Figure 4b and Figure 4c. Additionally, the values for V_O1_ and V_O2_ are almost the same, which means that they lead to the same structure. The structural dependency of the defective MoO_3_ monolayer is reflected in the insets of Figure 4b and Figure 4c. Previous theoretical studies show that a narrow and sharp peak in the electronic DOS around the Fermi level will generate a large transport distribution function and maximize the power by a factor of *S*^2^σ [24,35]. Especially for V_O3_, the sharp peak is more clear than for the other structures, yielding the best thermoelectric performance. Finally, O vacancies can act as shallow donors and destroy the crystal symmetry, thereby enlarging the carrier concentration.

## Conclusion

In summary, we have presented a comprehensive study of the thermoelectric properties of the MoO_3_ monolayer by first-principles calculations and Boltzmann transport theory. Our results indicate that the MoO_3_ monolayer exhibits better thermoelectric performance along the *x*-axis than along the *y***-**axis because of the strong anisotropic behavior of both the electrical and thermal conductivity. The *ZT* value along the *x*-axis reaches 0.72 at 300 K, which is much higher than the values obtained for other oxides. On the other hand, we find that the introduction of O vacancies is an efficient way to improve the thermoelectric performance of the MoO_3_ monolayer. Such an improvement can be attributed to a sharp peak in the electronic DOS, which leads to a large transport distribution function. The monolayer structure with the O vacancy at the O3 position possesses a maximum room temperature *ZT* value of 0.84 (*x*-axis) and 0.12 (*y*-axis). This work should provide helpful theoretical guidance that is relevant for possible thermoelectric applications of two-dimensional TMOs.

## Supporting Information

File 1 Crystal structures of MoO_3_ with oxygen vacancies.
