# Supplementary material for: Improvement of the thermoelectric properties of a MoO3 monolayer through oxygen vacancies
Source: Beilstein J Nanotechnol. 2019 Oct 25;10:2031–8. doi: 10.3762/bjnano.10.199 (PMC6839554; doi:10.3762/bjnano.10.199)
Supplement: File 1 — Crystal structures of MoO3 with oxygen vacancies. [file Beilstein_J_Nanotechnol-10-2031-s001.pdf]

BEILSTEIN JOURNAL OF NANOTECHNOLOGY

Supporting Information

for

Improvement of the thermoelectric properties of a MoO_3 monolayer through oxygen vacancies

Wenwen Zheng, Wei Cao, Ziyu Wang, Huixiong Deng, Jing Shi and Rui Xiong

Beilstein J. Nanotechnol. **2019,** *10,* 2031–2038. doi:10.3762/bjnano.10.199

Crystal structures of MoO_3 with oxygen vacancies

License and Terms: This is a supporting information file under the terms of the Creative Commons Attribution License (http://creativecommons.org/licenses/by/4.0). Please note that the reuse, redistribution and reproduction in particular requires that the authors and source are credited.
The license is subject to the *Beilstein Journal of Nanotechnology* terms and conditions: (https://www.beilstein-journals.org/bjnano)

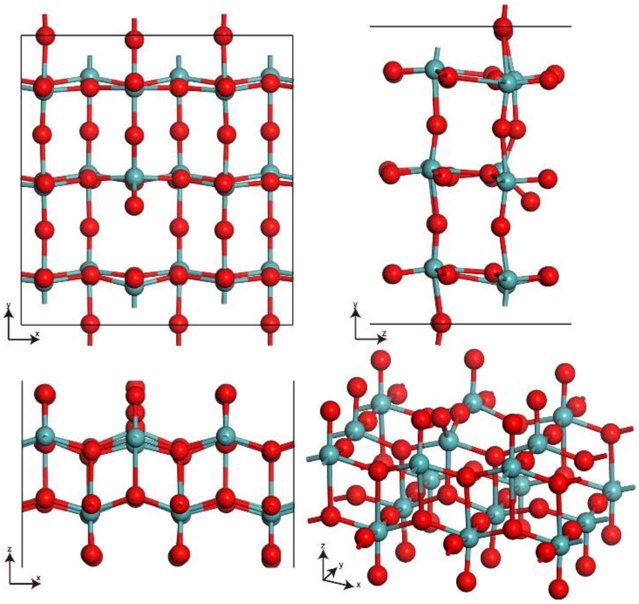

Figure S1: Optimized structure of MoO_3 monolayer with O1 vacancy. The red spheres represent O atoms and the green represent Mo atoms.

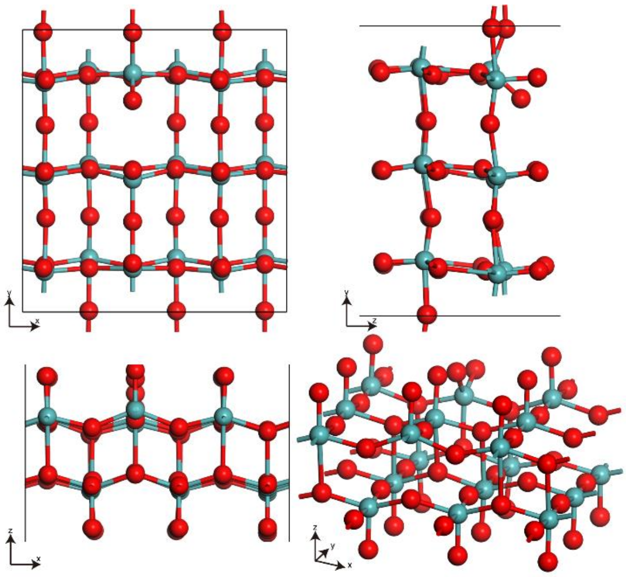

Figure S2: Optimized structure of MoO_3 monolayer with O2 vacancy. The red spheres represent O atoms and the green represent Mo atoms.

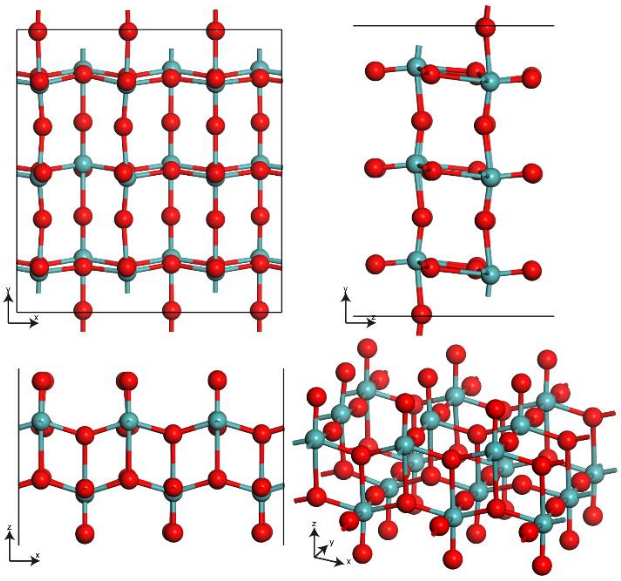

Figure S3: Optimized structure of MoO_3 monolayer with O3 vacancy. The red spheres represent O atoms and the green represent Mo atoms.
